# Supplementary material for: The meaning of momentary psychotic-like experiences in a non-clinical sample: A personality perspective
Source: PLoS One. 2022 Apr 20;17(4):e0267054. doi: 10.1371/journal.pone.0267054 (PMC9020697; doi:10.1371/journal.pone.0267054)
Supplement: S1 Table — (DOCX) [file pone.0267054.s001.docx]

Supplementary Material

https://osf.io/94t6p/
