# Supplementary material for: The meaning of momentary psychotic-like experiences in a non-clinical sample: A personality perspective
Source: PLoS One. 2022 Apr 20;17(4):e0267054. doi: 10.1371/journal.pone.0267054 (PMC9020697; doi:10.1371/journal.pone.0267054)
Supplement: Attachment — Submitted filename: letter_responses_esm_delta_after second_revision.docx [file pone.0267054.s003.docx]

Letter with responses

PLoS One journal

Manuscript: PONE-D-21-28042R1

Manuscript title: The meaning of momentary psychotic-like experiences in a non-clinical sample: a personality perspective

*Dear Dr. Luciano,*

*We would like to thank you and reviewers for the time and effort you invested in handling our manuscript. We would also like to thank you for overall positive feedback.*

*We have responded to the comment of Reviewer #2 and our responses are given in blue letters.*

*We hope you will find our manuscript suitable for publication in your journal.*

*Looking forward to receiving your response.*

*On behalf of co-authors,*

*Kind regards,*

*Goran Knezevic*

Reviewer #1: Review of PONE-D-21-28042R1, “The meaning of momentary psychotic-like experiences in a non-clinical sample: a personality perspective”

In my opinion this revised manuscript is ready for publication.

*Thank you very much for the positive evaluation of our manuscript. We would like to thank you for the thoughtful comments you made previously.*

Reviewer #2: The authors have been thoughtful in replying to my previous concerns, and the manuscript is significantly improved. I appreciate their clarifications.

*Thank you very much for positive feedback.*

My remaining concern is about the validation of the psychosis measure being used. It seems they have some data pertinent to this, in a paper under review by Ristić et al. (2021). It is difficult to evaluate the manuscript without this; as such, it seems best to present those analyses in the current paper, or wait until the other paper has been accepted for publication, or to refer to a preprint that might be available. In the very least, citing and discussing the Ristić et al. (2021) paper in the form of a preprint seems appropriate. Otherwise it's difficult to interpret the psychosis measure being used.

*We understand your concerns.*

A. *First, we added the following sentence in the footnote 5 in the manuscript (p. 16): “To further investigate validity of MPLEs we suggest to analyze correlations between thought disorders diagnosis and items from 20-item Delta scale, close to MPLEs (data based on the national representative sample of Serbian population,* https://osf.io/f8sje/*)”.*

*Now about the dataset we are referring to: The best data we have documenting the validity of DELTA scale/facets/items are recently collected as part of the epidemiological study investigating the influence of Covid-19 pandemic on mental health. The advantage comparing to Ristic et al. paper (2022) is that the Protocol paper of epidemiological study has been already published* (Maric et al 2021, <https://pubmed.ncbi.nlm.nih.gov/34548371/>), *and another paper is under review with higher chances to be published soon (see preprint* <https://psyarxiv.com/j3ac8>*). The data from this epidemiological study is more relevant than the data from Ristic’s et al. (2022) because in the latter the correlations between PANSS and DELTA were given only for those diagnosed as psychotic.*

*The findings obtained in the epidemiological study suggest strong validity of the 20-item version of D scale being used in this study. The version used in this epidemiological study was derived from the full, 110-items version used in our experience sampling study. The database - on which the findings we are showing you here are based - is publicly available (so everything can be verified)*: https://osf.io/f8sje/. *The sample (N=1203) on which epidemiological study was conducted is representative for the general population in Serbia (three-steps, random walk, household sample). Clinical diagnoses were established by using M.I.N.I. 7.0.2 by trained clinicians. Altogether, 183 individuals from our sample were diagnosed with at least one of 17 current psychiatric diagnoses. Out of them 22 were diagnosed as current psychotic (19) or current manic/hypomanic episodes (3) (i.e. 22 of those with HiTop thought disorder). As you can see in the Table 1 below, D total score, all facets, and all items (except for one) discriminate well between those diagnosed as thought disorder and those without any diagnosis (N=1020).*

| Table 1 |  | |  |  |
| --- | --- | --- | --- | --- |
|  | *Thought disorders*  *n=22* | *No disorders*  *n=1020* |  |  |
|  |  | | *Cohen's d* |  |
| *Disintegration, total score (D)* | *2.59 (0.85)* | *1.43 (0.55)* | *1.62**** | *Corresponding items in experience sampling -MPLEs* |
| *Facets* |  |  |  |  |
| *General executive/cognitive impairment (GE/CI)* | *2.64 (1.21)* | *1.54 (0.75)* | *1.09**** |  |
| *Perceptual distortions  (PD)* | *2.33 (1.18)* | *1.22 (0.56)* | *1.20**** |  |
| *Paranoia  (PAR)* | *2.80 (1.38)* | *1.72 (0.78)* | *0.96**** |  |
| *Apathy/Depression (A/DEP)* | *1.80 (1.07)* | *1.22 (0.55)* | *0.40**** |  |
| *Magical Thinking (MT)* | *2.82 (1.39)* | *1.44 (0.84)* | *1.20**** |  |
| *Enhanced Awareness (EA)* | *2.59 (1.50)* | *1.40 (0.77)* | *1.00**** |  |
| *Flattened Affect (FA)* | *3.05 (1.16)* | *1.50 (0.86)* | *1.52**** |  |
| *Somatoform dysregulation (SOD)* | *2.36 (1.19)* | *1.27 (0.62)* | *1.14**** |  |
| *Mania (MAN)* | *3.02 (1.25)* | *1.63 (0.92)* | *1.26**** |  |
| *Items* |  |  |  |  |
| *Sometimes, while doing something, I experience a mental block, because my mind turns blank (GE/CI)* | *3.14 (1.61)* | *1.34 (0.84)* | *1.41**** | *My thoughts were confused* |
| *I frequently repeat useless actions (GE/CI)* | *2.64 (1.62)* | *1.36 (0.86)* | *0.99**** |  |
| *On occasion, when I’m about to say something I end up saying something completely different (GE/CI)* | *2.14 (1.58)* | *1.93 (1.27)* | *0.15* |  |
| *Sometimes I feel as a split personality (PD)* | *2.50 (1.63)* | *1.29 (0.76)* | *0.96**** | *I had feeling I was not myself* |
| *I feel that everything around me is unreal  (PD)* | *2.27 (1.64)* | *1.18 (0.64)* | *0.88**** |  |
| *Sometimes I feel like someone else inside of me makes decisions instead of me  (PD)* | *2.23 (1.66)* | *1.18 (0.66)* | *0.83**** |  |
| *People speak ill of me (PAR)* | *3.18 (1.47)* | *2.25 (1.24)* | *0.68**** | *I had feeling that everyone was against me* |
| *I feel being watched  (PAR)* | *2.41 (1.71)* | *1.19 (0.68)* | *0.94**** |  |
| *I feel the presence of evil forces around me, although I can’t see them (MT)* | *3.27 (1.64)* | *1.43 (1.01)* | *1.36**** | *I felt presence of strange forces around me* |
| *I feel I can influence the course of events only by thinking a lot about them (MT)* | *2.36 (1.65)* | *1.45 (0.97)* | *0.67**** |  |
| *Sometimes I have an impression that my feelings are frozen (FA)* | *3.09 (1.54)* | *1.55 (1.07)* | *1.16**** | *I did not feel anything* |
| *It happens to me not no feel anything in a situation that should produce strong emotions (FA)* | *3.00 (1.66)* | *1.45 (0.92)* | *1.15**** |  |
| *I often wish I were dead and far away from everything (A/DEP)* | *1.36 (1.04)* | *1.12 (0.52)* | *0.29** | *I feelt that everything is pointless* |
| *I can’t find a will to do anything (A/DEP)* | *2.23 (1.77)* | *1.31 (0.78)* | *0.67**** |  |
| *Sometimes my body, or a part of my body, becomes insensitive and numb (SOD)* | *2.23 (1.63)* | *1.18 (0.63)* | *1.11**** | *My body did not function well* |
| *Sometimes I feel I will burst from the inside (SOD)* | *2.50 (1.57)* | *1.36 (0.88)* | *0.90**** |  |
| *Sometimes I think that many life rules don’t hold for me (MAN)* | *2.82 (1.56)* | *1.45 (0.95)* | *1.06**** | *I felt like almighty superman* |
| *I often feel happy but also irritable at the same time (MAN)* | *3.23 (1.51)* | *1.82 (1.21)* | *1.03**** |  |
| *Sometimes thoughts and pictures come to me all by themselves (EA)* | *2.64 (1.65)* | *1.34 (0.88)* | *0.98**** | *I felt that my mind can overwhelm the entire world* |
| *Someone’s voice can mesmerize me so much that I could go on listening to it forever (EA)* | *2.55 (1.68)* | *1.46 (1.00)* | *0.79**** |  |

*It is obvious that MPLEs in experience sampling study are constructed to represent the content of the facets closely. This means, if a 9 item measure had been constructed out of these items, it would likely have shown similar validity coefficients. As we already stated, the D model was a suitable theoretical framework because it suggests a wider range of experiences to be treated as psychotic-like than some alternative models of PLE (such as PID-5). We could have selected experiences such as hallucinations (in terms of D model, the most severe psychotic-like experiences are part of the D facets Perceptual Distortion and Somatoform Dysregulation), but it would have been far less convincing to demonstrate that such experiences were best predicted by D or PID-5 (ignoring the fact that extreme experiences would be rare in the student population). To demonstrate that hallucinations belong to PLE and not HEXACO space is not an epistemologically excited result, but to demonstrate that experiences that were not a part of the most influential contemporary PLE models (roughly, experiences related to Apathy/Depression, Mania, Flattened Affect, General Cognitive/Executive Impairments, Paranoia, Somatoform Dysregulations) belong to this space is an important result.*

*Meanwhile, four new articles have been published this year further testifying on the validity of Disintegration construct.*

Knežević, G., Savić, D., Vermetten, E., & Vidaković, I. (2021). From war-related trauma exposure to PTSD and depression: a personality perspective. *Journal of Research in Personality, 96,  104169.* [*https://doi.org/10.1016/j.jrp.2021.104169*](https://doi.org/10.1016/j.jrp.2021.104169)

Knežević, G., Lazarević, Lj., Bošnjak, M., & Keller, J. (2022). Proneness to psychotic-like experiences as a basic personality trait complementing the HEXACO model—A preregistered cross-national study. *Personality and Mental Health.* [*https://doi.org/10.1002/pmh.1537*](https://doi.org/10.1002/pmh.1537)

Knežević, G., Lazarević, Lj., Međedović, J., Petrović, B. & Stankov, L. (2022). The relationship between closed-mindedness and militant extremism in a post-conflict society. *Aggressive Behavior*, 48, 253-263. <https://doi.org/10.1002/ab.22017>

Stanković, S., Lazarević, Lj., & Knežević, G. (2022). The role of personality, conspiracy mentality, REBT irrational beliefs, and adult attachment in COVID-19 related health behaviors. *Studia Psychologica, Special issue on Psychological roots of questionable health practices. https://psyarxiv.com/q2nye/*

*Protocol paper:*

Marić, N. P., Lazarević, Lj.B., Mihić, L., Pejović Milovančević, M., Terzić, Z., Tošković, O., Todorović, J., Vuković, O. & Knežević, G., (2021). Protocol of the national representative survey of mental health in the second year after the COVID-19 outbreak: Multilevel analysis of individual and societal factors. *BMJ Open, 11(9):e053835. doi: 10.1136/bmjopen-2021-053835*

*Preprint of the paper, submitted to journal:*

Maric, N. P., Lazarevic, L., Priebe, S., Mihic, L., Pejovic-Milovancevic, M, Terzic-Supic, Z., Toskovic, O., Vukovic, O., Todorovic , J., & Knezevic, G. (2022)*. Covid-19-related stressors and mental disorders and distress: A cross-sectional, nationally-representative, face-to-face survey in Serbia. Preprint available at:* <https://psyarxiv.com/j3ac8>*.*
